# The Salicylic Acid-Mediated Release of Plant Volatiles Affects the Host Choice of *Bemisia tabaci*

**DOI:** 10.3390/ijms17071048

**Published:** 2016-06-30

**Authors:** Xiaobin Shi, Gong Chen, Lixia Tian, Zhengke Peng, Wen Xie, Qingjun Wu, Shaoli Wang, Xuguo Zhou, Youjun Zhang

**Affiliations:** 1 Department of Plant Protection, Institute of Vegetables and Flowers, Chinese Academy of Agricultural Sciences, Beijing 100081, China; xiaobin.s@163.com (X.S.); gongchen105@163.com (G.C.); tianlixia555@126.com (L.T.); zkpeng0827@163.com (Z.P.); xiewen@caas.cn (W.X.); wuqingjun@caas.cn (Q.W.); wangshaoli@caas.cn (S.W.); 2 Plant Protection Institute, Hunan Academy of Agricultural Sciences, Changsha 410125, China; 3 Department of Entomology, University of Kentucky, Lexington, KY 40546, USA; xuguozhou@uky.edu

**Keywords:** *Bemisia tabaci*, choice test, salicylic acid, plant volatile, plant–insect interaction

## Abstract

The whitefly *Bemisia tabaci* (Gennadius) (Hemiptera: Aleyrodidae) causes serious crop losses worldwide by transmitting viruses. We have previously shown that salicylic acid (SA)-related plant defenses directly affect whiteflies. In this study, we applied exogenous SA to tomato plants in order to investigate the interaction between SA-induced plant volatiles and nonviruliferous *B. tabaci* B and Q or B- and Q-carrying *tomato yellow leaf curl virus* (TYLCV). The results showed that exogenous SA caused plants to repel nonviruliferous whiteflies, but the effect was reduced when the SA concentration was low and when the whiteflies were viruliferous. Exogenous SA increased the number and quantity of plant volatiles—especially the quantity of methyl salicylate and δ-limonene. In Y-tube olfactometer assays, methyl salicylate and δ-limonene repelled the whiteflies, but the repellency was reduced for viruliferous Q. We suggest that the release of plant volatiles as mediated by SA affects the interaction between whiteflies, plants, and viruses. Further studies are needed to determine why viruliferous Q is less sensitive than nonviruliferous Q to repellent plant volatiles.

## 1. Introduction

The whitefly *Bemisia tabaci* (Gennadius) (Hemiptera: Aleyrodidae) is a highly polyphagous pest that causes substantial economic crop losses worldwide. In recent years, *B. tabaci* has rapidly adapted to new climatic zones and has expanded its host range [1]. *B. tabaci* damages crops by feeding on phloem sap and by transmitting viruses [2,3]. *B. tabaci* B and Q are the two most invasive whiteflies, and they have invaded nearly 60 countries during the last two decades [2].

In China, the concurrence of the spread of *tomato yellow leaf curl virus* (TYLCV) with the invasion of Q suggests a mutualistic relationship between TYLCV and Q; the relationship between TYLCV and B, in contrast, seems to be neutral or deleterious [4,5,6,7]. Based on these results, we hypothesize that plant defense may play an important role in the interaction of whiteflies with plants and viruses.

Plant protect themselves against herbivorous insects with both direct defenses, such as those involving plant hormones, and indirect defenses, such as those involving plant volatiles [8,9,10,11]. A recent study showed that the deposition of free salicylic acid (SA) on plants elicits a strong defense response against whiteflies [12]. Our previous results showed that B-carrying TYLCV induces more SA than Q-carrying TYLCV and that the performance of viruliferous B was reduced more than that of viruliferous Q on plants treated with exogenous SA [13]. Another study showed that aphid growth was greater on salicylate-deficient plants than on wild-type plants, and was lower on salicylate-induced plants than on non-induced control plants [14].

SA induces resistance in plants against biotrophic pathogens and some phloem-feeding insects [15,16,17]. Thus, feeding by aphids or whiteflies induces SA-related defenses [18,19,20]. Plant volatiles can also contribute to plant defense against herbivores and pathogens [21,22]. Although plant volatiles can help insects recognize host plants [23,24], volatiles can also attract predators and parasitoids in response to herbivore-induced damage [25,26,27,28]. A “push-pull strategy” of insect pest management has recently attracted increasing attention [29,30]. This strategy involves the use of stimuli that repel pests or prevent pests from locating crops and the use of stimuli that attract natural enemies or that attract pests to trap crops. Implementing a “push-pull” strategy obviously requires an understanding of which volatiles repel or attract herbivorous insects and their natural enemies. The role of SA in the production and release of plant volatiles that repel phloem-feeding insects is unknown.

We have previously reported that the application of exogenous SA to plants negatively affected nonviruliferous and viruliferous B and Q [13]. In the current study, we investigated how the exogenous application of SA affected the release of volatiles from the treated tomato plants and how the volatiles affected the choice of B and Q with and without TYLCV. We also used a Y-tube olfactometer to determine whether specific volatile compounds induced by SA repelled or attracted viruliferous or nonviruliferous B and Q. The results of this research will increase our understanding of the role of SA in plant defense against *B. tabaci* and TYLCV, and will also suggest novel strategies for increasing plant defense against *B. tabaci*.

## 2. Results

### 2.1. Establishment of Nonviruliferous and Viruliferous B. tabaci Colonies

**Figure 1:**
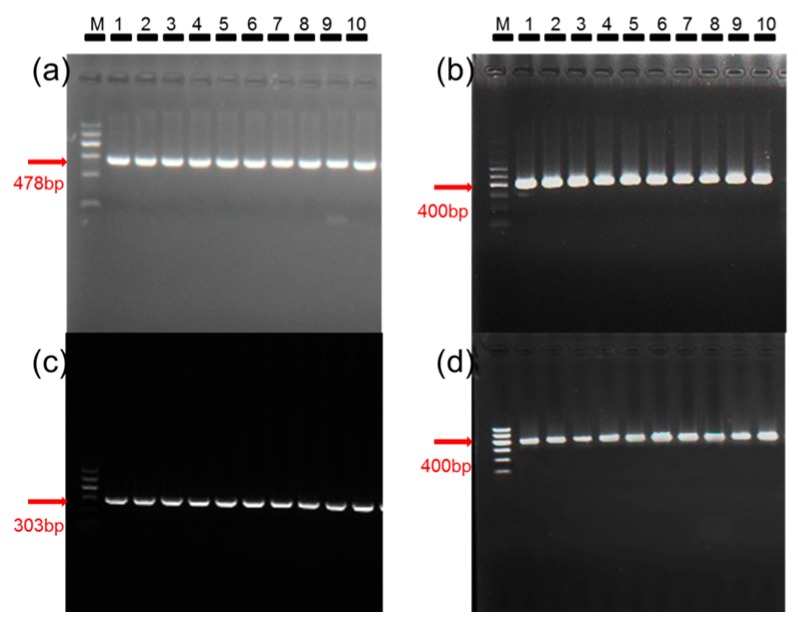
Confirmation of the status of nonviruliferous and viruliferous colonies of *B. tabaci* B and Q based on amplification of *mtCOI* for the whiteflies and on amplification of the *AV2* gene for *tomato yellow leaf curl virus* (TYLCV). M: marker; lane 1–10: whitefly individuals. In the case of nonviruliferous colonies, the amplified product had the correct size for B (478 bp, (**a**)) and for Q (303 bp, (**c**)). In the case of viruliferous colonies, the whitefly DNA was divided into two parts: one part was used to confirm whitefly identity and purity as described in the previous sentence; the other part was used to confirm the presence of TYLCV by amplification of the virus’s *AV2* gene. In each of the detection, the results of 10 biological replicates are shown in this figure. For viruliferous whiteflies, the amplified product had the correct size for B and Q as indicated in (**a**,**c**), and an amplification product of 400 bp was obtained for both viruliferous B (**b**) and viruliferous Q (**d**), indicating that B and Q whiteflies in the viruliferous colony contained TYLCV.

The identity and purity of the colonies of B and Q used in this study were confirmed by using the molecular diagnostic technique CAPS (cleavage amplified polymorphic sequence) and the molecular marker *mitochondrial cytochrome oxidase I* gene (*mtCOI*) (Figure 1a,c). The presence of TYLCV in the viruliferous colonies and its absence in the nonviruliferous colonies was confirmed by amplification of the *AV2* gene (Gene ID: 949226) (Figure 1b,d).

### 2.2. Choice Test of B. tabaci on SA-Treated vs. Control Plants

**Figure 2:**
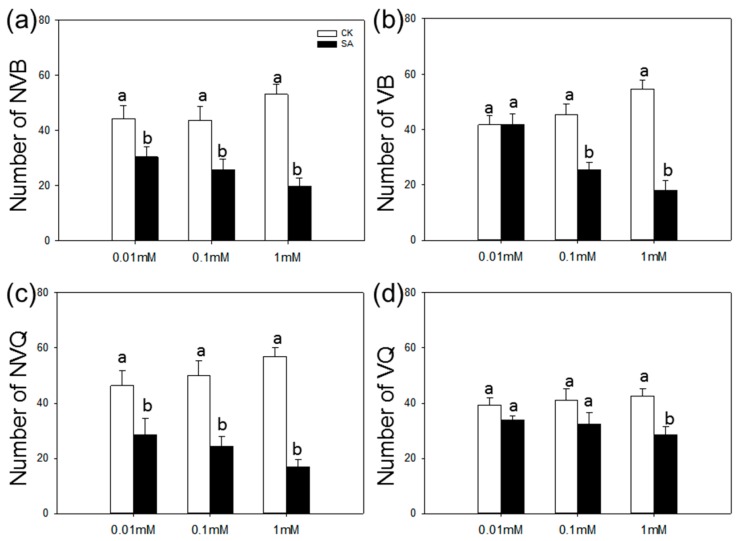
Choice test of *B. tabaci* on SA-treated vs. control plants. Choice test of nonviruliferous B (**a**); viruliferous B (**b**); nonviruliferous Q (**c**); and viruliferous Q (**d**) as indicated by numbers per plant. Three concentrations of SA were used: 0.01, 0.1, and 1 mM. Values are means ± SE (*n* = 9). Different lowercase letters indicate significant differences between control plants and SA-treated plants (LSD test at *p* < 0.05).

The choice test of nonviruliferous B and Q (NVB and NVQ, respectively) and viruliferous B and Q (VB and VQ, respectively) for SA-treated and control plants was investigated. The numbers per plant were significantly lower on SA-treated plants than on control plants in all cases (NVB: *F* = 249.437, *p* < 0.001; NVQ: *F* = 120.761, *p* < 0.001; VB: *F* = 459.871, *p* < 0.001; VQ: *F* = 10.360, *p* = 0.007) (Figure 2). The number of whiteflies was not significantly affected by three SA concentrations (NVB: *F* = 2.572, *p* = 0.118; NVQ: *F* = 0.267, *p* = 0.770; VB: *F* = 0.003, *p* = 0.997; VQ: *F* = 0.103, *p* = 0.903). However, the number of *B. tabaci* that selected SA-treated plants tended to decrease as SA concentration increased (Figure 2b).

### 2.3. Extraction and Analysis of Volatiles

The quantities and identities of volatiles released by tomato plants were significantly affected by the application of exogenous SA (Table 1). The following compounds were released in significantly higher concentrations by SA-treated plants than by control plants: α-pinene, β-phellandrene, ρ-cymene, and α-humulene. The other two volatiles, methyl salicylate and δ-limonene, were released from the SA-treated plants but not from the control plants (Table 1).

### 2.4. Effects of Methyl Salicylate and δ-Limonene on Whitefly Behavior as Determined with a Y-Tube Olfactometer

**Figure 3:**
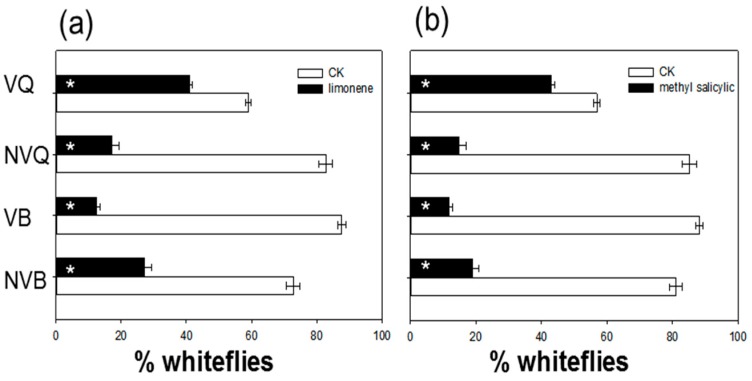
Choice test of nonviruliferous and viruliferous *B. tabaci* B and Q for (**a**) δ-limonene vs. control volatiles and for (**b**) methyl salicylate vs. control volatiles in a Y-tube olfactometer. NVQ: nonviruliferous Q; VQ: viruliferous Q; NVB: nonviruliferous B; VB: viruliferous B. Values are means ± SE (*n* = 9). Asterisks indicate significant differences (*p* < 0.05).

In a Y-tube olfactometer, a lower percentage of the whiteflies moved into the arm with methyl salicylate than without methyl salicylate, and the percentage was also affected by virus status but not whitefly species (Figure 3, Table 2). Fewer whiteflies also moved into the arm with δ-limonene than without δ-limonene, but the percentage was not affected virus status or whitefly species (Figure 3, Table 2). The percentage of viruliferous Q that moved into the arm with methyl salicylate or δ-limonene was greater than the corresponding percentages of nonviruliferous Q, nonviruliferous B, or viruliferous B.

## 3. Discussion

Herbivorous insects can use plant volatiles to locate hosts and to obtain information about host condition [31]. Although plant volatiles may indirectly prevent the insects from colonizing the plant by attracting natural enemies to the site of herbivory [32,33,34], they can also attract or resist herbivorous arthropods directly [35,36]. Plant volatile manipulated by insects can make the plants attractive or repellent to other herbivores [37]. Horiuchi et al. (2003) [38] reported that when lima bean plants are slightly infested by two-spotted spider mite, they can attract the conspecific mites, whereas when they are heavily infested, they can repel the conspecifics. Aphid infestation may induce secondary metabolites of host plants that deter *B. tabaci* from settling on the same host plants [39]. Common armyworms (*Mythimna separata*) can utilize plant volatiles emitted from maize plants infested by conspecifics to determine their nocturnal behavior [40].

SA is involved in activating a number of plant responses to herbivory including the production and release of volatiles [41]. Plants treated with exogenous SA reduced the fecundity and longevity of nonviruliferous and viruliferous *B. tabaci* B and Q [13]. However, how SA affects plant volatiles and host choice of insects is largely unknown. Our results showed that exogenous SA repelled nonviruliferous *B. tabaci* B and Q. Viruliferous Q, however, was not repelled by plants sprayed with 0.01 or 0.1 mM SA concentration. Previous results showed that whiteflies can decrease the emission of volatiles by plants [42]. We therefore speculate that viruliferous Q was not greatly repelled by tomato plants treated with a low SA concentration perhaps because viruliferous Q may manipulate the emission of volatiles triggered by SA.

The oral secretions and saliva enzymes of insects can help to explain this speculation. Plant volatiles are mediated by a complicated network of signal perception and transduction. The oral secretions and saliva enzymes of insects have been reported to participate in this process. For example, when tobacco plants are infested by *Manduca sexta*, fatty acid amino acid conjugates were found in the oral secretions, which were introduced into plant wounds during *M. sexta* feeding [43]. Ca^2+^ influx has been reported to function in gel saliva enzymes of aphid elicitors (e.g., oligogalacturonides) [44], but there is still a missing link between the biosynthetic pathways and the induced volatiles. In our research, the oral secretions or saliva enzymes of *B. tabaci* Q may be changed by TYLCV, and plant volatile may be induced differently during the feeding of viruliferous Q. Furthermore, the olfactory sensilla of *B. tabaci* Q may also be altered by TYLCV. These speculations need to be further investigated.

The changes in volatiles released by SA-treated tomato plants may have altered the choices of whiteflies for treated vs. non-treated plants. Plant volatiles include a wide range of active substances such as terpenes and green leaf volatiles, which can function as airborne signals within and between plants [31]. The quantities of the volatiles α-pinene, β-phellandrene, ρ-cymene, and α-humulene released by tomato plants were increased by SA treatment, and all four volatiles are terpenes. Terpenes play a major role in mediating plant–herbivore interactions [45,46,47,48]. We conclude that SA activates multiple pathways involved in the synthesis of tomato volatiles.

In the current study, the volatiles methyl salicylate and δ-limonene were released from SA-treated plants but not from the control plants. Many reports have shown that the ester methyl salicylate and the monoterpene δ-limonene can be induced by herbivore attack and may help defend against herbivore attack [49,50,51,52]. Limonene of the citrus peel has been shown to confer partial resistance in fruits to the Mediterranean fruit fly, medfly (*Ceratitis capitata*), a major pest of citrus species worldwide [53]. MeSA has been reported to be involved in plant defence, particularly in the elicitation of systemic acquired resistance (SAR) [54]. Although methyl salicylate was not one of the volatiles collected from control tomato plants in the current study or in two previous studies [55,56], it has been detected as a volatile released by tomato plants in other studies [57,58,59]. Perhaps this discrepancy is due to differences in the cultivars used in the studies.

When given a choice between δ-limonene or methyl salicylate and purified air in a Y-tube olfactometer assay, most of the nonviruliferous and viruliferous whiteflies in the current study preferred purified air, i.e., they were repelled by the two volatiles (Figure 2). The repellency by δ-limonene and methyl salicylate, however, was much lower for viruliferous *B. tabaci* Q than for nonviruliferous Q or for viruliferous or nonviruliferous B. This was consistent with the results of the assay with SA-treated tomato plants in which viruliferous Q showed no preference for treated vs. untreated plants when the SA concentration was low (Figure 1d). A possible explanation is that TYLCV alters the oral secretions, saliva enzymes, or olfactory sensilla of viruliferous Q. Further research is needed to determine why viruliferous Q exhibits reduced sensitivity to SA-induced volatiles.

## 4. Materials and Methods

### 4.1. Host Plants

Tomato plants (*Lycopersicon esculentum*, cv. Zhongza 9) were grown in a glasshouse. TYLCV-infected tomato plants were produced at the 3–4 true leaf stage by *Agrobacterium tumefaciens*-mediated inoculation with a cloned TYLCV genome (GenBank accession ID: AM282874) that was originally isolated from Shanghai, China [60]. Viral infection of test plants was confirmed by the development of characteristic leaf curl symptoms and by molecular analysis of tomato leaves with the TYLCV primer set *TYLCV-61* and *TYLCV-473*, which amplified the *AV2* gene (Gene ID: 949226) [5].

### 4.2. Establishment of Nonviruliferous and Viruliferous B. tabaci Colonies

*B. tabaci* B and Q were originally collected from infested cabbage (*Brassica oleracea*. cv. Jingfeng 1) and poinsettia (*Euphorbia pulcherrima* Wild. ex Klotz.) in fields in Beijing, China [5]. We created four whitefly colonies: nonviruliferous B, nonviruliferous Q, viruliferous B, and viruliferous Q. Viruliferous colonies were created by placing four TYLCV-infected tomato plants in each of two cages (60 × 60 × 60 cm). Then, 300 nonviruliferous B and Q adults were transferred to each of the two cages, one biotype per cage. Nonviruliferous B and Q colonies were simultaneously established by placing 300 nonviruliferous B and Q adults in cages with virus-free tomato plants, one biotype per cage. All colonies were maintained for more than six generations in separate greenhouses at 25 ± 1 °C, 60% ± 100% Relative Humidity and Light14: Dark10. The purity of these colonies was monitored by sampling 20 adults per generation using the molecular diagnostic technique CAPS (cleavage amplified polymorphic sequence) and the molecular marker *mitochondrial cytochrome oxidase I* gene (*mtCOI*) [61]. The presence of TYLCV in the viruliferous whiteflies was confirmed by molecular analysis of DNA of the individual 30 whiteflies with the TYLCV primer set *TYLCV-61* and *TYLCV-473*, which amplifies the *AV2* gene (Gene ID: 949226) [5]. For the latter determination, the viruliferous whitefly DNA was divided into two parts: one part was used to determine the whitefly biotype, and the other part was used to detect TYLCV.

### 4.3. Effect of Salicylic Acid (SA) Application on Tomato Plants

SA (Sigma Chemical Co., St. Louis, MO, USA) was dissolved in ethanol (1 mL) and dispersed in water containing 10% Tween 20 to produce a 1 mM SA solution. The 1 mM SA solution was diluted to prepare 0.1 mM and 0.01 mM SA solutions. A hand-sprayer was then used to treat the foliage of healthy tomato plants (with six true leaves) with 0.01, 0.1, or 1 mM SA. As a control, other plants were sprayed with water (containing 1 mL ethanol and 10% Tween 20). Each of the four treatments was represented by 9 plants.

### 4.4. Choice Test of B. tabaci on SA-Treated vs. Control Plants

Tomato plants of the same size were treated with water or with one of the three concentrations of SA as described in the previous section. Two plants (one treated with SA and one control) were placed 40 cm apart in opposite corners of a whitefly-proof screen cage (80 × 40 × 60 cm). At 7:00 a.m. on the day of the experiment, a suction tool was used to collect about 100 adult whiteflies (either *B. tabaci* B or Q and either nonviruliferous or viruliferous that had been starved for 24 h) in a clear centrifuge tube. After the centrifuge tube with whiteflies was placed in the center of the cage, the lid of the tube was removed so that the whiteflies could fly out of the tube and approach the plants from above. The number of whiteflies on each treated or control plant was determined after 48 h. To prevent whiteflies from being disturbed and from relocating to the other plant during counting, each plant was enclosed in transparent plastic and was assessed at 7:00 a.m. Each choice test experiment was performed in 9 cages (3 concentrations of SA × 3 technical replications) at one time, and the experiment was performed three times.

### 4.5. Extraction and Analysis of Volatiles

Volatiles emitted by control tomato plants or tomato plant treated with 1 mM SA were collected using a headspace collection system as described by Zhang et al. [62] with slight modification. After volatiles were trapped for 6 h under continuous light, the plants were weighed (FW) so that the quantity of volatile released could be expressed per g of FW. Each of the two treatments was represented by 9 replicate plants.

Headspace samples were dissolved in *n*-hexane that contained 0.2 μg·mL^−1^ of *n*-dodecane as an internal standard. Then, a 1-μL sample of the supernatant was subjected to gas chromatography–mass spectrometry. The temperature profile was as follows: 50 °C 1 min; 50 to 240 °C, 5 °C·min^−1^; 240 °C 2 min; 240 to 300 °C, 30 °C·min^−1^; and 300 °C 5 min. The injection temperature was 270 °C. The temperature of the source was 200 °C, and the interface temperature was 280 °C. The column effluent was ionized by electron impact ionization (70 eV). Compounds were identified by comparison of GC retention times and normalization of peak areas with those of internal standard and true standards whenever possible, and by comparison of mass spectra with spectra of the NIST database. When standards were not available, the concentrations of the compounds were matched to published information [63,64].

### 4.6. Y-Tube Olfactometer Assays

Choice tests of whiteflies for the plant volatiles methyl salicylate and δ-limonene (which were the volatiles whose concentrations were most increased by SA application) were determined in a Y-tube olfactometer. Two streams of purified air (filtered through activated charcoal) were passed through two glass containers (one containing methyl salicylate or δ-limonene and the other containing purified air) and into the olfactometer arms at 100 mL·min^−1^. Each assay began with the release of one adult whitefly at the base of the Y-tube. Each whitefly was observed for a maximum of 20 min. A “no choice” was recorded when the adults remained inactive during the assay period, and a choice for one of the two odor sources was recorded when the whitefly moved >5 cm onto either arm and stayed in that arm for at least 15 s. For each of the two volatiles, four whitefly populations were tested: nonviruliferous B, viruliferous B, nonviruliferous Q, and nonviruliferous Q. For each population, the preference of 30 whiteflies was tested three times in 1 day. The experiment was then repeated on two additional days. As a consequence, a total of 270 whiteflies were tested for each of four populations.

### 4.7. Statistical Analysis

All proportional data were arcsine-square root transformed before analyses. Concentrations of volatiles emitted from SA-treated and control tomato plants were compared with *t*-tests. Whitefly choice for plants that were treated with control vs. SA (at three concentrations) was compared with the General Linear Model (GLM). The choice of whiteflies for methyl salicylate or δ-limonene vs. control in the Y-tube olfactometer was also compared with the General Linear Model (GLM). SPSS version 20.0 (SPSS Inc., Chicago, IL, USA) was used for all statistical analyses.

## 5. Conclusions

Our results confirm that plant volatiles mediated by SA play an important role in the interaction of whiteflies with plants and viruses. SA applications could be used to repel whiteflies, and it could also be used to reduce whitefly fecundity, longevity, and survival on target crops [13]. SA applications could also increase the quantity and makeup of volatiles released by plants, resulting in reduced choice behavior of whiteflies. Exogenous SA had a reduced effect on viruliferous Q. Further studies will be needed to investigate why viruliferous Q has an increased ability to resist host defenses such as plant volatiles.

## Figures and Tables

**Table 1:** Concentrations (µg·g·FW^−1^) of volatile compounds released from tomato plants treated or not treated (control) with salicyclic acid (SA) ^a^.

Volatile Compound	Control Plants ^b^	SA-Treated Plants ^c^
3-Hexenal	0.422 ± 0.112 A	0.561 ± 0.098 A
3-Hexen-1-ol	0.130 ± 0.012 A	0.376 ± 0.070 A
α-Pinene	0.029 ± 0.007 A	0.245 ± 0.027 B
β-Myrcene	0.819 ± 0.197 A	1.826 ± 0.864 A
β-Phellandrene	0.089 ± 0.037 A	0.513 ± 0.099 B
β-Caryophyllene	0.939 ± 0.303 A	1.612 ± 0.072 A
ρ-Cymene	0.325 ± 0.069 A	1.188 ± 0.092 B
α-Phellandrene	2.935 ± 0.096 A	2.485 ± 0.151 A
α-Humulene	0.073 ± 0.020 A	0.307 ± 0.037 B
Methyl salicylate	n.d.	3.428 ± 0.141
δ-Limonene	n.d.	2.420 ± 0.146

^a^ Values are means ± SE. Volatiles were compared by horizontal not vertical direction. For each compound, different uppercase letters (A and B) indicate significant differences between SA-treated plants and control plants (LSD test at *p* < 0.05). n.d.: not detected; ^b^ Plants were sprayed with water (containing 1 mL ethanol and 10% Tween 20) 24 h before volatiles were trapped; ^c^ Plants were sprayed with 1 mM SA 24 h before volatiles were trapped.

**Table 2:** *p*-Values from two-way ANOVA analysis concerning the percentage of nonviruliferous and viruliferous B and Q whose movement was affected by methyl salicylate or δ-limonene in a Y-tube olfactometer.

Independent Variable	Dependent Variable ^a^	Independent Variable	Dependent Variable
Methyl salicylate	<0.001 ***	δ-Limonene	<0.001 ***
V ^b^	0.045 *	V	0.542
Biotype ^c^	0.884	Biotype	0.542
Methyl salicylate × V	<0.001 ***	δ-Limonene × V	0.225
Methyl salicylate × Biotype	<0.001 ***	δ-Limonene × Biotype	<0.001 ***
V × Biotype	<0.001 ***	V × Biotype	0.189
Methyl salicylate × V × Biotype	<0.001 ***	δ-Limonene × V × Biotype	<0.001 ***

^a^ Significance levels are indicated by * *p* < 0.05; and *** *p* < 0.001; ^b^ whitefly virus status (nonviruliferous and viruliferous); ^c^ whitefly biotype (B and Q).
